# Design of a Biologically Inspired Water-Walking Robot Powered by Artificial Muscle

**DOI:** 10.3390/mi13040627

**Published:** 2022-04-15

**Authors:** Dongjin Kim, Minseok Gwon, Baekgyeom Kim, Victor M. Ortega-Jimenez, Seungyong Han, Daeshik Kang, M. Saad Bhamla, Je-Sung Koh

**Affiliations:** 1 Department of Mechanical Engineering, Ajou University, San 5, Woncheon-dong, Yeongtong-gu, Suwon 443-749, Korea; rlaehdwlswt@ajou.ac.kr (D.K.); pj4990@ajou.ac.kr (M.G.); www7541@ajou.ac.kr (B.K.); sy84han@ajou.ac.kr (S.H.); dskang@ajou.ac.kr (D.K.); 2 School of Chemical and Biomolecular Engineering, Georgia Institute of Technology, Atlanta, GA 30332, USA; ornithopterus@gmail.com

**Keywords:** biologically inspired robots, SMA actuator

## Abstract

The agile and power-efficient locomotion of a water strider has inspired many water-walking devices. These bioinspired water strider robots generally adopt a DC motor to create a sculling trajectory of the driving leg. These robots are, thus, inevitably heavy with many supporting legs decreasing the velocity of the robots. There have only been a few attempts to employ smart materials despite their advantages of being lightweight and having high power densities. This paper proposes an artificial muscle-based water-walking robot capable of moving forward and turning with four degrees of freedom. A compliant amplified shape memory alloy actuator (CASA) used to amplify the strain of a shape memory alloy wire enables a wide sculling motion of the actuation leg with only four supporting legs to support the entire weight of the robot. Design parameters to increase the actuation strain of the actuator and to achieve a desired swing angle (80°) are analyzed. Finally, experiments to measure the forward speed and angular velocities of the robot are carried out to compare with other robots. The robot weighs only 0.236 g and has a maximum and average speed of 1.56, 0.31 body length per second and a maximum and average angular velocity of 145.05°/s and 14.72°/s.

## 1. Introduction

Biologically inspired robotic systems provide a deeper understanding of highly efficient and multi-functional organisms in nature. Their unique principles inspire novel robotic mechanisms showing a comparable or even higher performance than conventional robotic systems. For example, the flapping flight on an insect scale [1], jumping on water inspired by a water strider [2], swimming inspired by a diving beetle [3] and crawling hexapod inspired by a cockroach [4] demonstrate unique locomotion and the high potential of biologically inspired robotic systems. Notably, water striders have been a significant inspiration source for the development of microrobots that can walk on water [5,6,7]. The surface-tension-driven locomotion of water striders due to scaling effects results in little resistance to motion. Thus, water striders have an agile motion with peak speeds of 1.5 m/s. Mimicking the biological functions of a water strider could enable a water-walking robot to perform aquatic tasks, such as water quality monitoring and searching even in shallow water.

To develop a mechanical water strider that can move on water with highly agile motion and less drag, many studies have developed water-walking robots that perform sculling motion on water. Since Hu et al. [8] first built a device that can row on a water surface with elastic energy storage, various water-walking robots [9] driven by DC motors were introduced. Yan et al. [10,11,12] proposed miniature robots based on a cam-link mechanism. Zhao et al. [13] fabricated a robot utilizing a latch-and-spring-based actuating mechanism that releases high energy for propulsion. Directly utilizing the rotational motion of a DC motor without any complex linkages, Zhang et al. [14] designed a coiled actuating leg and Song et al. [15] designed an untethered robot with actuating legs in a rectangular shape. These coiled and rectangular legs were directly connected to DC motors. Ozcan et al. [16] showed an untethered robot with circular footpads and a crank-rocker mechanism. Suzuki et al. [17] developed two water-walking robots driven by a vibration motor and a slider-crank mechanism. These DC motor-based water-walking robots are inevitably heavy because of the weight of the actuator and accessory components to achieve the desired wide range motion of the actuating legs. To support the heavy weight of the actuator and additional parts, DC motor-based robots require up to 12 long supporting legs or wide footpads to increase the lift force. The additional supporting legs and wide footpads increase resistance in motion, especially in the turning motion.

There are studies that employ other types of actuators, such as piezoelectric actuators [18,19] and shape memory alloy (SMA) actuators [20]. The piezoelectric and shape memory alloy actuator are suitable candidates for water-walking robots considering their lightweight. However, they also have limitations in actuation strain and power consumption. Piezoelectric actuators exhibit a light weight and high actuation frequency, but a small motion of the leg driven by the actuator results in a low velocity of the robot. Even though SMA is considered to provide a low actuation strain, it has a higher range of strain than that of a piezoelectric actuator. The SMA actuator could achieve a large leg motion similar to that of the water strider, provided that a proper amplification mechanism is utilized. Therefore, a careful selection of the actuator is important to achieve high performance in the velocity of the water-walking robot. A robot with a suitable actuator should be as light as possible with similar morphology to real water striders, such as the number of supporting legs and the ability to perform a wide sculling motion of the actuating leg.

**Figure 1:**
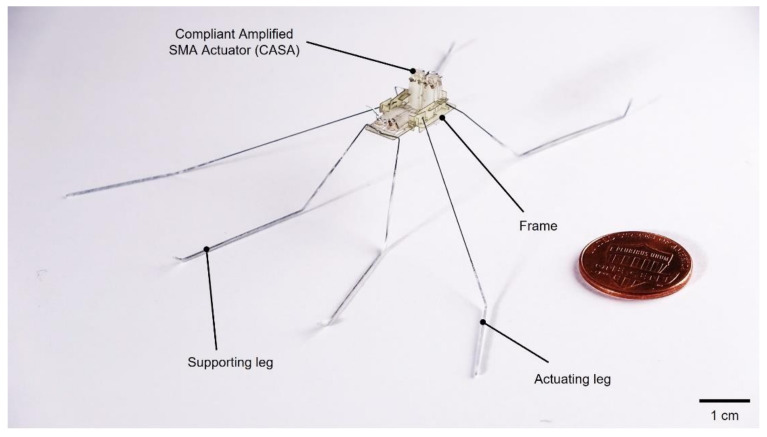
Image of a water-walking microrobot.

In this study, we applied a light SMA-based actuator providing actuation power strong enough for a water-walking microrobot to move on water. The SMA-based water-walking robot is capable of moving forward and turning with four degrees of freedom (Video S1). The design of an amplification mechanism to employ an SMA wire is discussed to achieve the highest actuation strain of the actuator. With the compliant amplified SMA actuators (CASAs), the sculling mechanism is developed to generate a desirable trajectory of the actuating leg. A final robot prototype (Figure 1) is manufactured, and experiments are conducted to test its performance, which are compared to other water-walking robots. This research provides an example of a surface-tension-driven micro aquatic robot that can serve as a platform for studies of the locomotion mechanism of the water strider.

## 2. Materials and Methods

### 2.1. Fabrication

**Figure 2:**
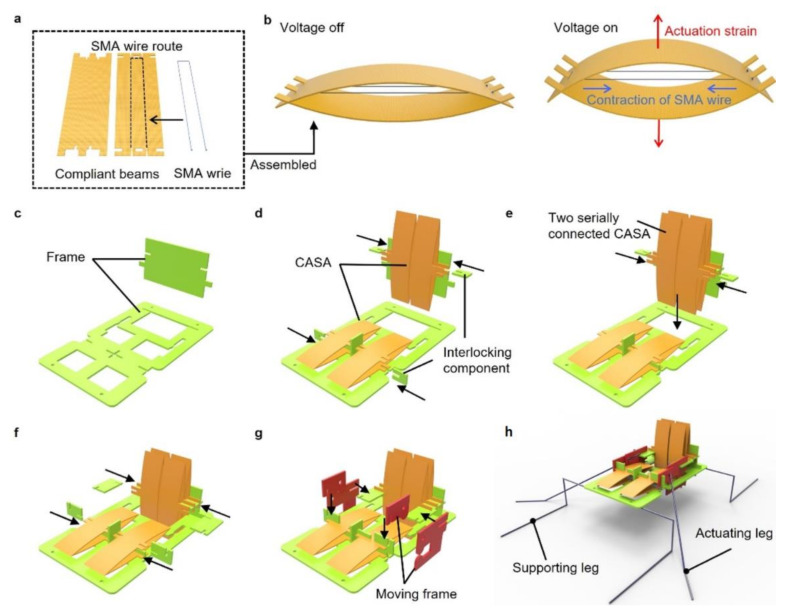
Fabrication of an actuator and a robot. (**a**) Components of a compliant amplified SMA actuator (CASA); (**b**) Schematic of an assembled CASA actuating with voltage on and off; (**c**–**h**) fabrication steps for a robot composed of fixed frames, actuators and moving frames connected by interlocking component, supporting legs and actuating leg.

In this section, we discuss the design of the water-walking robot and the SMA-based actuator to produce the sculling motion. A previously designed SMA-based actuator [21] amplified low strain of an SMA wire with a compact form and lightweight due to its simple structure. The compliant amplified SMA actuator (CASA) consisted of compliant beams (glass-reinforced epoxy laminate sheet) and the SMA wire (DYNALLOY, Inc., Irvine, CA, USA), as shown in Figure 2a. Two crimps were attached at the ends of SMA wire. The crimps were anchored at the compliant beams when the SMA wire was placed through orifices of laser-machined compliant beams. The assembled actuator had a bow-like shape where the tension of the SMA wire and buckling force of the compliant beams were at equilibrium, as shown in Figure 2b. When a voltage was applied to the SMA wire, the phase transition occurred, resulting in a contraction of the SMA wire. Then, the contraction in horizontal direction caused the beams to bend and we utilized this amplified vertical bending motion for the actuation of the water-walking robot.

To achieve the light weight of the robot, we fabricated a body of the robot with only laser-cut glass-reinforced epoxy laminate sheet (FR-4) with a thickness of 0.2 mm. The body consisted of fixed frames, CASAs, moving frames and components that interlocked each component. Figure 2c shows that two perpendicular frames were assembled. Each frame served as the ground for the actuators to operate in perpendicular directions, as shown in Figure 2d. The actuator could be stacked serially for a higher stroke (Figure 2e). Moving frames were then interlocked with CASAs, which moved vertically and horizontally, as shown in Figure 2f,g. Finally, supporting legs were attached to the fixed frame and each actuating leg was placed through two holes in the separate moving frames as shown in Figure 2h. The legs were super elastic Nitinol wire with diameter of 0.2 mm. The legs were coated with a superhydrophobic coating (Ultra-Ever Dry 4000, 4001). The moving frames penetrated by the actuating leg enabled a rotation of the actuating leg when the moving frame was moved by the CASAs. The fabricated robot only weighed 0.236 g due to the lightweight actuators and the sheet-type material used to develop the sculling mechanism.

### 2.2. Design of the CASA

**Figure 3:**
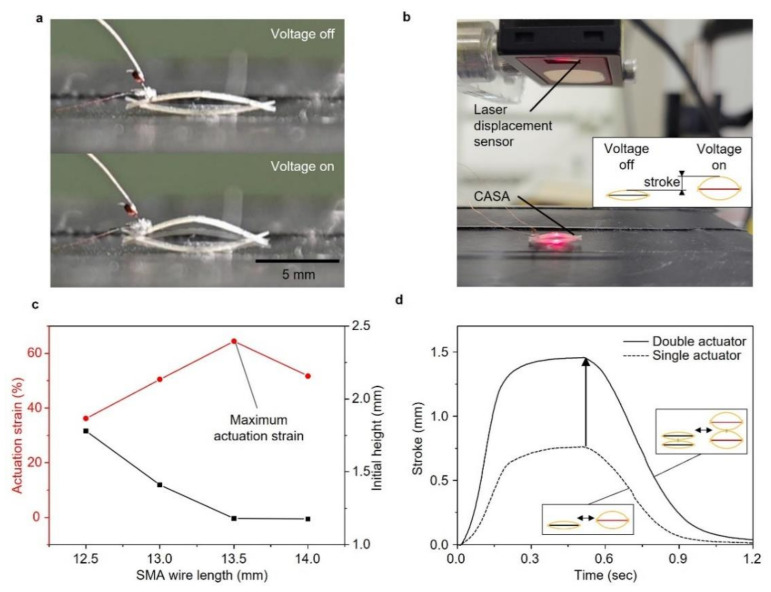
Design and performance of a CASA. (**a**) Image of actuation of a CASA; (**b**) experimental setup to measure actuation strain of a CASA; (**c**) actuation strain for different length of SMA wire length; (**d**) actuation stroke of a single actuator and a stack of double actuators.

The compliant structure of the CASA amplified small strain (3~4%) of the SMA wire when the voltage was applied, as shown in Figure 3a. Previous research [21] on the CASA demonstrated that a prestrain of SMA wire and initial height of assembled elastic beams determined the actuation strain of the CASA. Highest actuation was achieved with these two key parameters. First, the prestrain of SMA wire in an elastic deformation and the actuation strain of the SMA wire in the horizontal direction were in a proportional relationship. A short SMA wire resulted in the high prestrain caused by bending force of the compliant beams; thus, the high actuation strain. The second parameter was initial the height of compliant beams. The low initial height induced high incremental ratio of the vertical-to-horizontal displacement. A long SMA wire caused the compliant beams to bend with a low initial height; thus, the high amplifying rate. It was important to find the optimal length of the SMA wire for two inversely proportional parameters. We tested four different lengths of the SMA wire to find the highest actuation strain of the CASA.

We used a laser displacement meter (Panasonic HG-C1030-P) to measure the actuation strain of the CASA, as shown in Figure 3b. We used 12.5 mm, 13 mm, 13.5 mm and 14 mm length of SMA wire to fabricate the CASAs and measure the actuation strain (Figure 3c). The result showed that the optimal length of SMA wire for sufficient prestrain with low initial height was 13.5 mm long SMA wire. The CASA with the 13.5 mm long SMA wire actuated with 64.51% of maximum amplified actuation strain. The CASAs with maximum actuation strain could also be serially stacked to multiply the stroke. The stroke of the single CASA was measured 0.761 mm, as shown in Figure 3d. We fabricated two serially connected CASAs with 1.456 mm of stroke. We employed the single and double-stacked actuators to produce sculling trajectory of the actuating leg as explained in detail below.

### 2.3. Design of Sculling Mechanism

**Figure 4:**
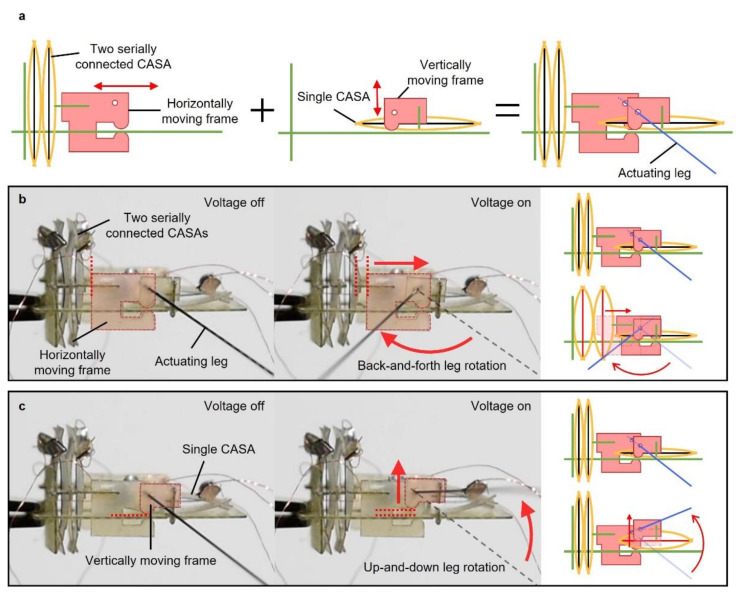
Image and illustration of a side view of robot showing rowing mechanism. (**a**) Schematics of vertically and horizontally moving components; (**b**) first degree of freedom, back-and-forth actuating leg rotation; (**c**) second degree of freedom, up-and-down actuating leg rotation.

Separate or simultaneous motions of the water strider’s middle legs enable the water strider to move forward or to turn. The robot had four actuators (two single and two stacked CASAs) to create the separate sculling motion of each actuating leg. Figure 4 shows a side view of the robot. There were two different sets of an actuator and moving frame designed to move in two different perpendicular directions, as shown in Figure 4a. The actuating leg was placed through two holes of two moving frames. Each moving frame was designed to move only in one direction different from each other, in the horizontal and vertical direction. The actuating leg rotated back and forth as the horizontally moving frame moved and the other vertically moving frame was, fixed serving as a pivot (Figure 4b). To move the horizontally moving frame, two serially stacked CASAs were connected to the moving frame. Figure 4b demonstrates how the actuating leg rotated up and down. The single CASA was connected to the vertically moving frame. As the vertically moving frame was moved by the single CASA connected to vertically moving frame, the actuating leg rotated up and down with the horizontal moving frame fixed. At each side of the robot, the horizontally and vertically embedded actuators could perform the motion with 2 degrees of freedom. Since the actuators at each side were separated, the total degree of freedom of the robot was 4. The back-and-forth rotation angle was designed about two times higher than the up-and-down rotation angle to achieve similar elliptical trajectory of the leg of a real water strider. The combination of rotation of the actuating leg in two different directions created the sculling motion explained in detail in the Section 3.

**Figure 5:**
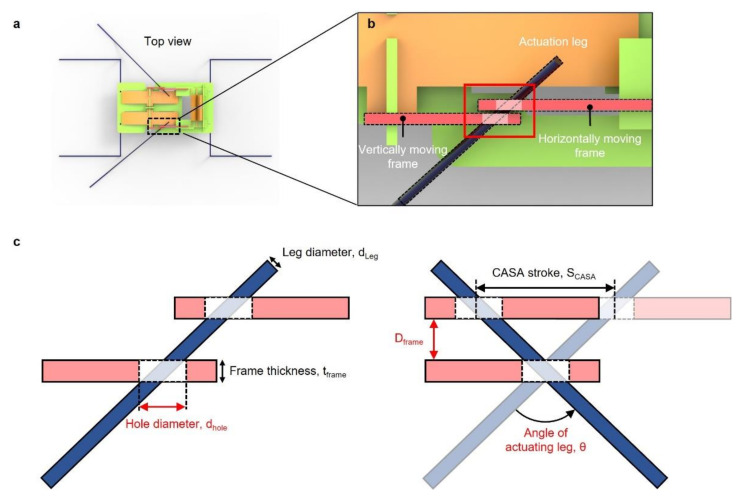
Design parameters for desirable angle of actuating leg. (**a**) Schematic top view of a robot; (**b**) enlarged schematic noted as dashed rectangle in top-view schematic; (**c**) schematic of moving frames and actuating leg before and after actuation with parameters for desirable angle of an actuating leg.

The water strider could swing its middle leg for propulsion and the horizontal swing range of the leg was approximately from −45° to 30° [22]. According to this swing range, the sculling mechanism of our robot was designed to achieve the horizontal swing angle of 80°. Figure 5a shows the top view of the robot and enlarged image in Figure 5b describes how moving frames and actuating leg were arranged. To achieve desired 80° of the horizontal swing angle, the hole diameter, $d_{hole}$, and the distance between the moving frames, $D_{frame}$, needed to be determined. The hole diameter and the distance between the moving frames were expressed as:

(1) $$d_{hole}=\frac{t_{frame}}{\cot\frac{\theta }{2}}+\frac{d_{leg}}{\cos\frac{\theta }{2}}$$

(2) $$D_{frame}\leq \frac{S_{CASA}}{2\tan\frac{\theta }{2}}-t_{frame}$$

where $t_{frame}$ is the thickness of the frame (0.2 mm), $d_{leg}$ is the diameter of the actuating leg (0.2 mm), $S_{CASA}$ is the stroke of the CASA (1.456 mm) and θ is the desired angle of the actuation leg (80°), as shown in Figure 5c. $d_{hole}$ and $D_{frame}$ were calculated to be 0.429 mm and 0.6676 mm, respectively.

## 3. Results and Discussion

### 3.1. Trajectory of the Actuating Leg and Water-Walking Robot

**Figure 6:**
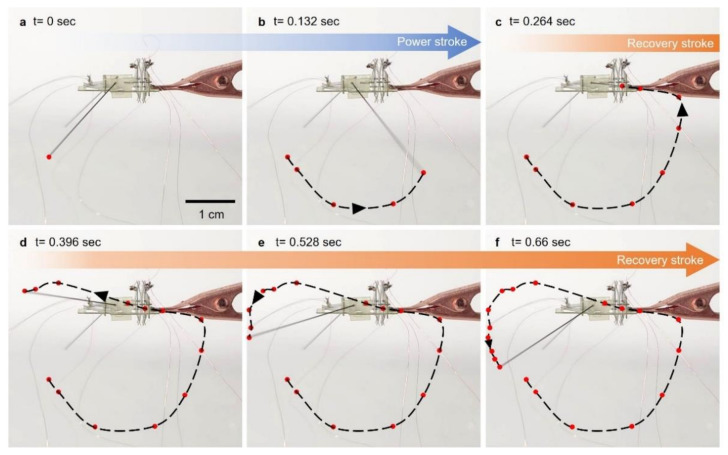
(**a**–**f**) Photo snapshots of rowing actuating leg trajectory in the air.

**Figure 7:**
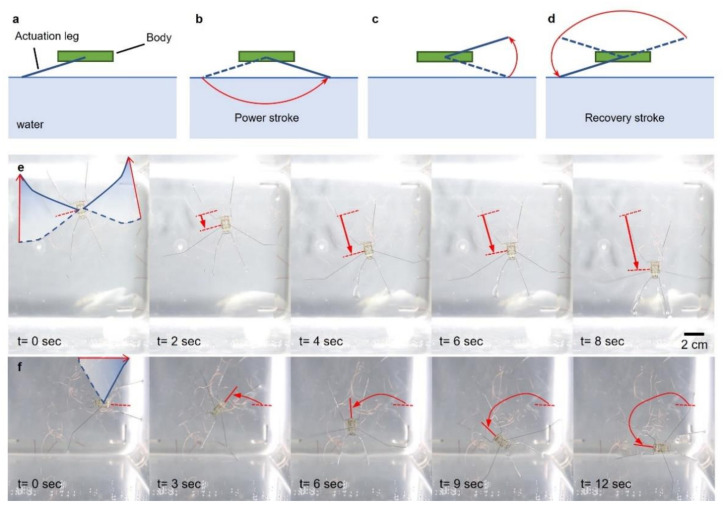
Image of a water-walking microrobot. (**a**–**d**) Sculling motion on the water; photo snapshots of robot (**e**) moving forward and (**f**) turning.

The robot was designed to achieve at least an 80° angle of a horizontal swing by the actuator connected to the horizontally moving frame. The robot also had an actuator for the vertical motion of the leg. Stimulating each actuator in sequence created a sculling motion of the leg, as shown in Figure 6. From the initial state in Figure 6a, the actuator for the horizontal motion actuated, enabling the leg to rotate backwards as shown in Figure 6b. This motion on the water generated a propulsion, as shown in Figure 7a,b. Right after swinging the leg backwards, the other actuator for the vertical motion actuated, causing the leg to rotate upwards as shown in Figure 6c. This motion enabled the leg to be lifted from the water (Figure 7c) for recovery to the initial state without touching the water. After the leg was lifted from the water, the actuator for the horizontal motion was turned off, returning to the initial state due to the elastic beams. The elastic beams were counter spring to return the SMA wire to the initial state without input energy. During the horizontal motion, the actuator was off, but the other actuator for the vertical motion was still on. The resulting trajectory is shown in Figure 6d. When the actuator for the horizontal motion returned to the initial state, the other actuator was turned off to lower the leg, as shown in Figure 6e,f. Turning off the actuator in sequence allowed for the recovery stroke above the water, as shown in Figure 7d. This cycle of actuation of the actuators enabled the robot to swing its actuation leg repeatably on water to continuously move on the water.

Our robot was capable of moving forward and turning by swinging two actuating legs simultaneously or only one actuating leg. Figure 7e demonstrates the forward motion of the robot swinging the actuating leg simultaneously. The frequency of the cycle for the sculling motion was 0.67 Hz. The frequency of the cycle was relatively low compared to other types of actuators. This was due to the required cooling time of the SMA wire. It took at least 600 ms to cool down the SMA wire upon returning to its initial state. Figure 7f shows that the robot was able to turn by swinging only one leg with another leg resting on the water. The actuating leg on the right side was actuated to turn the robot to the left.

### 3.2. Comparison with Other Robots

**Figure 8:**
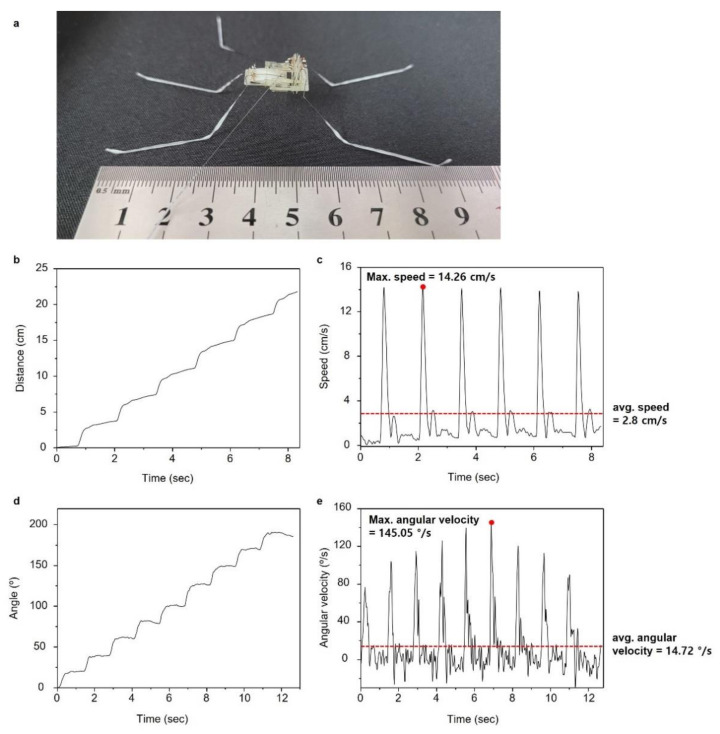
Experimental results of forward and turning motion. (**a**) Image of the robot indicating its body length (9 cm); (**b**) distance during forward motion of a robot; (**c**) velocity of the robot moving forward; (**d**) turning angle during turning motion of a robot; (**e**) angular velocity of the robot.

The distance and the speed of the robot moving forward were measured, as shown in Figure 8. The robot was 9 cm long, as shown in Figure 8a. The robot could move a distance of approximately 5 cm per stroke with the maximum and average speed of 14.26 cm/s (1.56 body length (BL) per second) and 2.8 cm/s (0.31 BL per second), respectively, as shown in Figure 8b,c. Even though a thin copper wire with a diameter of 0.01 mm was used for the electric connection, the wire interfered with the motion of the robot, reducing its speed due to the light weight of the robot. The turning, angle and maximum angular velocity of the robot were also measured (Figure 8d,e). The robot could turn approximately 20° per stroke with a maximum and average angular velocity of 145.05°/s and 14.72°/s, respectively.

**Figure 9:**
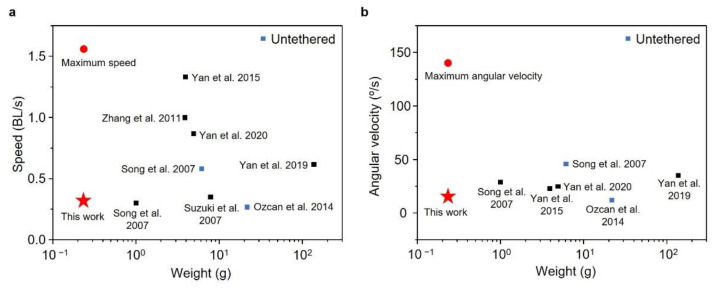
Comparison of weight, speed and angular velocity of water-walking robots. (**a**) Average speed and weight; (**b**) average angular velocity and weight.

We also compared the performance of our robot with other water-walking robots (Figure 9a,b). The current prototype exhibited a comparatively light weight (0.236 g), maximum speed (1.56 BL/s) and angular velocity (145.05°/s) and an average speed (0.31 BL/s) and angular velocity (14.72°/s). The use of the SMA wire and simple structure for the sculling motion reduced the weight of the robot. Including only four thin supporting legs to support its light weight reduced the resistance to motion. The SMA is known to actuate under low frequency compared to other types of actuators. Even though the time for a cycle of SMA actuation was long due to its cooling (recovering) time, the heating time could be greatly reduced by applying a high voltage. Thus, the robot utilizing the CASAs had the advantage of generating a highly propulsive energy in a short time, resulting in the high maximum speed. However, the robot’s average speed was comparatively low due to the long cooling time (low frequency) of the SMA wire. The untethered robots noted as a blue dot in Figure 9 were also proposed, weighing 6.13 g and 21.75 g. Our robot was expected to weigh less than 1 g with a battery and electronics to control the robot with on-board power.

## 4. Conclusions

In this paper, a light water-walking robot driven by an SMA-based actuator was developed. The amplification for the SMA wire was achieved by bow-like compliant beams. The optimal length of the SMA wire enhanced the actuation strain of the actuator. Employing single and double-stacked CASAs with suitable design parameters enabled the actuation leg to perform a wide sculling motion. The robot was capable of moving forward and turning by controlling two separate actuation legs. Our robot represented a steerable water-walking robot with maximum and average speeds of 1.56 BL/s and 0.31 BL/s, and maximum and average angular velocities of 145.05°/s and 14.72°/s.

Future work should include the development of a hydrodynamic model for the robot sliding on water, increasing the actuation frequency by employing a thinner SMA wire and fabricating the on-board powered robot. It is our hope that this robot serves as a platform to deepen the understanding of the locomotion of arthropods in the aquatic environment.

## Data Availability

Not applicable.

## Supplementary Materials

The following supporting information can be downloaded at: https://www.mdpi.com/article/10.3390/mi13040627/s1, Video S1: Moving forward and turning locomotion of a water strider and a water walking robot.
